# Profiling the B cell immune response elicited by vaccination against the respiratory virus SARS-CoV-2

**DOI:** 10.3389/fimmu.2022.1058748

**Published:** 2022-11-24

**Authors:** Elena Pettini, Donata Medaglini, Annalisa Ciabattini

**Affiliations:** Laboratory of Molecular Microbiology and Biotechnology, Department of Medical Biotechnologies, University of Siena, Siena, Italy

**Keywords:** memory B cells, SARS-CoV-2, COVID-19 vaccines, respiratory virus, computational flow cytometry, B cell ELISpot, BCR repertoire

## Abstract

B cells play a fundamental role in host defenses against viral infections. Profiling the B cell response elicited by SARS-CoV-2 vaccination, including the generation and persistence of antigen-specific memory B cells, is essential for improving the knowledge of vaccine immune responsiveness, beyond the antibody response. mRNA-based vaccines have shown to induce a robust class-switched memory B cell response that persists overtime and is boosted by further vaccine administration, suggesting that memory B cells are critical in driving a recall response upon re-exposure to SARS-CoV-2 antigens. Here, we focus on the role of the B cell response in the context of SARS-CoV-2 vaccination, offering an overview of the different technologies that can be used to identify spike-specific B cells, characterize their phenotype using machine learning approaches, measure their capacity to reactivate following antigen encounter, and tracking the maturation of the B cell receptor antigenic affinity.

## Introduction

The immune responsiveness to vaccination with a T cell dependent antigen is characterized by the induction of the B cell response, featured by the rapid generation of plasma cells, which release antigen-specific immunoglobulins with a peak 4-6 days after immunization, and the progressive induction of a memory long-lasting B cell response, capable of persisting months or years in the host (1).

Since the SARS-CoV-2 virus emerged in late 2019 much effort has been directed to the characterization of the immune response elicited by the infection or the vaccination, to understand possible differences, the duration over time, and the capability of recognizing new variants of SARS-CoV-2 that arise in the population.

Vaccination against SARS-CoV-2 has shown some peculiarities, since i) it was a mass vaccination across different ages, ii) it was implemented during the acute phase of the pandemic, iii) it has seen the use for the first time of new technologies such as RNA-based vaccines (2). These multi-factors have made understanding the immune mechanisms induced by vaccination particularly challenging. Confounding factors have been recognized in race, gender, age, comorbidities and immunosuppressive drug treatments (3). For example, age and immunological disorders, which can induce physiological and premature immune senescence, respectively, as well as chronic systemic low-grade inflammation have been considered among the most impacting factors on immune responsiveness to vaccination (4–8).

**Figure 1:**
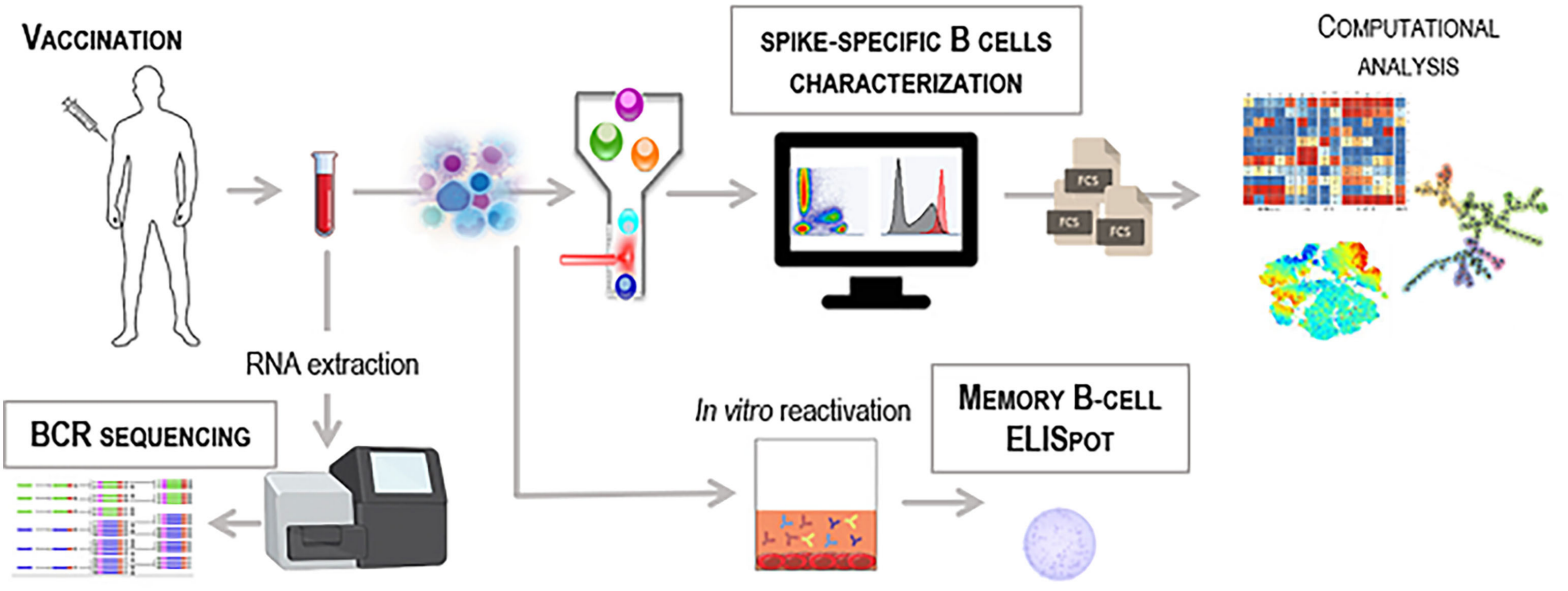
Workflow for the identification of vaccine-induced spike-specific B cells. Schematic representation of different technologies to detect antigen-specific B cells in blood samples collected from SARS-CoV-2 vaccinated subjects. PBMC can be used for multiparametric flow cytometry analysis labelling cells with the fluorescent antigen as probe to identify rare antigen-specific B cells. Computational flow cytometry can be then applied to explore and visualize multiparametric flow cytometry data (fcs files) using specific tools and algorithms. Effector function of spike-specific memory B cells can be measured in PBMC by B-cell ELISpot technique upon *in vitro* restimulation of cells. High-throughput bulk RNA sequencing of BCR heavy-chain genes can be performed on whole blood to unravel the dynamics of the BCR repertoire.

Most of the attention on the COVID-19 vaccination was initially focused on the vaccine *efficacy*, a measure of the degree to which a vaccine prevents disease calculated by comparing a vaccinated group with a placebo group. The first data on Moderna and Pfizer mRNA COVID-19 vaccines showed a promising efficacy around 95% (9, 10). Data on how well vaccines performed in the real world, vaccine *effectiveness*, were accumulated in the months after the beginning of the vaccination campaigns (11–13). Many works have thus correlated the protective capacity of SARS-CoV-2 vaccines with the induction of antibodies and in particular neutralizing antibodies (14, 15). Analysis of antibody persistence, however, demonstrated a physiological decline over time, with a greater drop in the first two months following the administration of the second vaccine dose (16). The decrease in circulating antibodies correlated with an increase of SARS-CoV-2 infection among vaccinated subjects (15, 17), prompting regulatory agencies to promote booster doses of vaccination order to raise the amount of circulating antibodies. The effect of the third booster dose proved particularly effective in some cohorts of fragile subjects, in whom the response to the first vaccination cycle had appeared slower and less intense (5, 6, 8, 18, 19). The booster dose also demonstrated the ability to reinvigorate the entire antibody response, also significantly increasing the levels of antibodies cross-reactive with new circulating variants of concerns, such as Delta and Omicron (18). Despite the antibody response is capable of inducing immediate protection, from the most severe forms of the disease and death (20), we should carefully not overlook the characterization, persistence and profiling of the memory B cell response capable of promoting new waves of antibody-secreting cells following contact with the virus. The increasing knowledge of immune mechanisms, together with a growing interest on the characterization of the T cell response, has contributed to a growing focus on the study of the cellular response and its persistence (21, 22). Here, we will provide insights into the role of the B cell response elicited by SARS-CoV-2 vaccination, and the different technologies that can be used to identify vaccine-induced spike-specific B cells, characterize their phenotype, also through computational approaches, assess their persistence, measure their effector capacity to reactivate following antigen encounter, and track the maturation of their antigenic affinity (**Figure 1**).

## Role of the B cell response elicited by vaccination

While a large amount of data has been produced to characterize the antibody response, the literature aimed at characterizing the B cell response to vaccination against SARS-CoV-2 remains rather limited. The study of COVID-19 vaccine immune responsiveness has become more complicated over time, as the characterization and monitoring of protective correlates elicited by vaccination have mixed with those induced or boosted by the natural infection, and it is unclear how to discriminate between the two. In fact, it has been observed an increase in hybrid immunity, in subjects who have been both infected with and vaccinated against SARS-CoV-2. This ‘interference’ of viral infection, *inter alia* with new circulating variants, on the dynamics of the vaccination-induced response is now the main focus of many studies (23–25). Besides a robust SARS-CoV-2-specific antibody response (26, 27), mRNA-based vaccines induce a robust class-switched memory B cell (MBC) response that is further enhanced after the second dose (26, 27). The induction and persistence of spike-reactive GC B cells within the draining lymph nodes, 30 weeks after primary vaccination with an mRNA vaccine has been demonstrated in a clinical study (28). Individuals previously infected with SAR-CoV-2 and then vaccinated with mRNA-based vaccine showed a significant increase in the spike-specific MBC (26, 27). The magnitude of this increase strongly correlates with the number of pre-existing SARS-CoV-2 MBC, indicating that MBC are critical in driving a recall response upon re-exposure to SARS-CoV-2 antigens (26). Other studies have reported that no further increase in the SARS-CoV-2-specific antibody or memory B cell response was observed upon administration of a second dose to previously infected individuals, suggesting that only one dose of the mRNA-based vaccine is necessary to reach peak humoral immunity in previously infected individuals (26, 29). It has also been shown that SARS-CoV-2-specific MBC evolve and mature over several months by the progressive acquisition of somatic mutations in their variable region genes and a sizable proportion of such memory cells is able to neutralize all variants of concern (25), except Omicron (30). Nevertheless, subjects receiving three doses of an mRNA vaccine have a diverse memory B cell repertoire that can respond rapidly and produce antibodies capable of clearing even diversified variants such as Omicron (17).

A time-dependent modulation of the B cell response has been observed, with B cell phenotypes changing from CD27^+^ IgD^+^ IgM^+^ CXCR5^+^ cells to both IgA^+^ or IgG^+^ (CD27^+^IgD^-^ CD38^+^CXCR5^-^CD11c^+^) 7 days after the second dose, and then differentiating into MBC (CD27^+^ IgD^-^ CD38^+^CXCR5^+^CD24^+^CD11c^-^) at longer time points (31). Several distinct antigen-specific MBC populations emerged postvaccination with varying kinetics. Spike-specific cells were IgG^+^ with a mature and resting CD21^+^ CD27^+^ phenotype (32).

The B cell response has been recently characterized also in fragile patients such as hematopoietic cell transplantation recipients, myelofibrosis patients and people living with HIV, immunized with mRNA vaccines (6, 8, 33). These studies have highlighted the crucial role of the third dose in increasing the frequency of spike-specific B cells, generating a response similar to the one detected in healthy controls.

## Assays to measure the B cell immune responses

### Flow cytometric identification of spike-specific B cells

**Figure 2:**
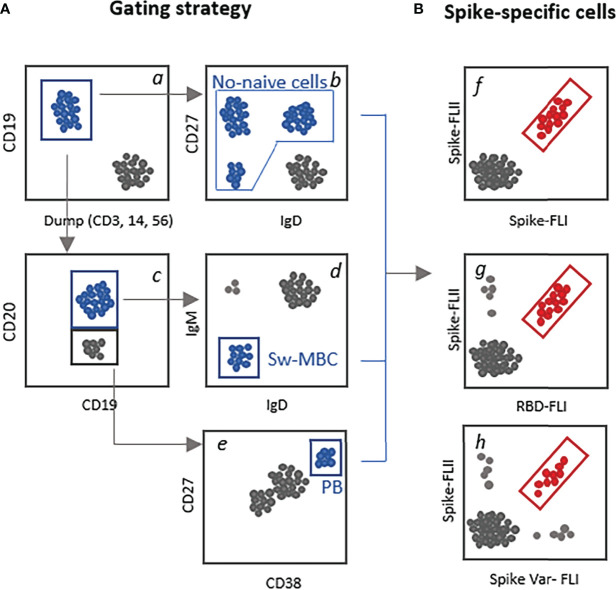
Schematic gating strategy for the identification of spike-specific B cells. **(A)** Examples of different strategies that can be used for selecting the parent subset of B cells to be analyzed for the presence of spike-specific B cells. Starting from CD19^+^ B cells gated among live, singlet cells *a*, it can be selected the not-*naïve* B cells by excluding the IgD^+^CD27^-^ cells *b*; the CD19^+^CD20^+^ *c*, switched MBC (IgD^-^IgM^-^, *d*, and the CD19^-/low^ CD20^-^ cells c CD38^high^CD27^+^ plasmablasts *e*. **(B)** Identification of antigen-specific-B cells using two fluorescent labelled spike proteins *f*, the full spike *versus* the RBD domain only *g* or *versus* the spike protein of a viral variant (var1, *h*).

Flow cytometric analysis allows the identification of single cells present in an heterogenous suspension allowing the identification of rare antigen-specific cells. In the case of SARS-CoV-2 the biotinylated spike protein, or its receptor binding domain (RBD), can be used as probes to bind all antigen-specific B cells. A subsequent step of tetramerization with fluorescent streptavidin molecules allows detection by flow cytometric analysis. To increase the specificity of binding, two fluorescently labelled RBD probes can be simultaneously used (34). It is also possible to use the full spike or S1 or S2 subunits towards the RBD domain only, to distinguish spike protein-specific clones outside the RBD region. Two-dimensional analysis of spikes versus RBD positive cells will allow the discrimination of RBD-specific clones (double-positives cells) from those recognizing spike domains present in the S2 region or part of S1 outside the RBD region (32). To identify MBC cross-reactive against variants of concerns, variant specific spike probes can be used in the staining panel (35). A schematic gating strategy for identification of B cells subsets and spike-specific B cells is reported in **Figure 2**.

Since flow cytometric analysis allows the simultaneous detection of numerous markers, this permits not only to identify and quantify spike-specific cells but also to study their phenotype and changes in molecules expression according to immune response maturation. All major populations of human peripheral B cells can be identified using a panel of surface markers including CD19, CD20, CD21, IgD, CD27, CD38, and CD24 (36).

Using multiparametric flow cytometry analysis, we and other groups have identified spike-specific cells in the blood induced by vaccination with mRNA BNT162b2 vaccine and we have tracked their persistence up to 6 months after vaccine administration (31, 32). The presence of molecules specific for different states of antigen-specific B-cell differentiation, allows to identify and distinguish plasmablasts, detectable very early after vaccine administration, from MBC. Modulation of surface immunoglobulin expression also allows us to study isotypic switching by assessing the expression of IgA or IgG on the cell surface. The presence of unswitched cells can be identified within CD27^+^IgD^+^ cells, which in most cases include IgD^+^ IgM^+^ double-positives cells. Plasmablasts, on the other hand, can be identified among the CD19^low^ CD20^-^ cells, as positive for CD38 and CD27. Detection of plasma cells, the terminal stage of differentiation of plasmablasts, remains more difficult due to certain functional and technical aspects, such as the lack of expression of surface BCR, since their fate is to release soluble antibodies to a specific antigen already recognized and selected. In this case, antigen-specific cell detection, mediated by fluorescent proteins, loses sensitivity. Moreover, cryopreservation of PBMC could induce substantial loss in marker expression and function of cells, due to osmotic activity that harms the cells during the cryopreservation and thawing process. This can impact of the surface detection of some markers, such as for example the CD138, one of the most specific molecule for plasma cells identification (37). To optimize detection of this molecule, staining of fresh cells is recommended (32).

### Machine learning analysis of flow cytometry data

Flow cytometric analysis of cell subsets has traditionally been performed with “manual gating” based on the measurement of two parameters visualized on bi-dimensional plots. This approach is simple and intuitive however, it constitutes a big source of variability, and when many parameters are investigated, is not feasible to visualize all the possible bi-dimensional combinations of marker expression. To overcome these limitations, novel computational techniques have been developed in recent years (38), and computational flow cytometry has been applied in the vaccine field, to characterize different B and T cell subsets and their functionality (39–41).

The workflow for the automated analysis of cytometric data includes pre-processing, automated analysis with data-visualization and result interpretation, each based on the use of specific computational tools (i.e. FlowJo plugins, web services, and libraries for some of the most common programming languages such as R and Python) (41). The two most used approaches to explore and visualize data are dimensionality reduction and unsupervised clustering. The first one allows displaying high-dimensional data in a lower-dimensional space, using two or three surrogate dimensions where each cell is represented as a dot, as the t-SNE method (42). This approach has been used for example for visualizing the trajectory of SARS-CoV-2 vaccine-induced immunity over the course of primary two-dose vaccination and after the third dose (43). Samples, clustered based on antibody and memory B cell responses using uniform manifold approximation and projection (UMAP), showed that naïve and COVID-recovered individuals clustered apart from each other at the pre- vaccination and early time points following the primary vaccination cycle, while began to converge in UMAP space at later memory time points and were indistinguishable after the third vaccine dose (43).

Algorithms based on an unsupervised clustering approach stratify cells with similar marker profiles in clusters, which can subsequently be interpreted as cell populations. FlowSOM is considered one of the best high-performance algorithms in automated identification of cell subsets showing an extremely fast runtime (44). Unsupervised clustering analysis has been applied for identifying major B cell populations and SARS-CoV-2–specific B cells elicited by the mRNA vaccines and determining their modulation at the different time points following vaccine administration (31, 32). The analysis has allowed the identification of different spike-specific B cell subsets, including naïve cells, unswitched and isotype-switched MBC and plasmablasts. Correlation between specific clusters of cells and humoral response is also possible; the analysis has demonstrated that the frequency of plasmablasts detected 7 days after the second vaccine dose positively correlated with the frequency of IgG^+^ switched MBC clusters measured at day 180 (31, 32).

### B cell ELISPOT

In peripheral blood samples it is possible to detect antibody secreting cells (ASCs) directly *ex vivo*, or to identify antigen-specific MBC by restimulating peripheral blood mononuclear cells (PBMC) to drive their differentiation into ASCs. Owing to its high sensitivity, enzyme-linked immune absorbent spot (ELISpot) assay is especially useful for detecting and enumerating discrete populations of active cells (i.e. antigen-specific cells) and represents therefore an important complement to conventional serology in profiling B cell responses to vaccination. The B cell ELISpot has proven to be an important method for both the detection of IgG-producing B cells (45) and antigen-specific memory B cells (46, 47). Whereas ASCs can be examined directly without *in vitro* activation, MBC require a pre-stimulation step with an antigen-independent polyclonal activator in order to promote the differentiation into ASC (48). When performing ELISpot, the timing of the blood collection should be carefully chosen, since it has been previously demonstrated that antigen-specific ASC peak in peripheral blood around day seven post immunization and then rapidly decrease to become undetectable after 2 weeks, while functional MBC increase from 3 to 6 months post vaccination and then persist at a steady level for long periods of time after immunization as circulating antigen-experienced B cells.

The ELISpot technique has been successfully employed to quantify spike-specific ASC or memory B cells induced by SARS-CoV-2 vaccination. We and others have studied the frequency of spike-specific antibody-secreting cells in PBMC upon restimulation to quantify SARS-CoV-2 specific memory MBC response up to 6 months following mRNA vaccination (26, 31, 49, 50). The ELISpot has also been used to determine the presence of cells secreting IgG or IgA antibodies against spike protein in bone marrow aspirates collected 29 weeks after vaccination, that were detected in 82% of patients (28). Liu and colleagues characterized spike- and RBD-specific MBC upon inactivated SARS-CoV-2 vaccine administration, showing that MBC persisted despite a decreasing trend over a 5-month time frame between second and third immunization, and that the third dose significantly increased the antigen-specific MBC (51).

### BCR repertoire

Next-generation sequencing approaches to study B cell receptor (BCR) heavy chain repertoires have been used for the dynamic characterization of the B cell response, at the level of individual B cell clones, elicited by SARS-CoV-2 vaccination. Following antigen exposure further diversification of the repertoire occurs, through somatic hypermutation and subsequent selection of high-affinity clones within the germinal centers (52).

Deep sequencing supported by state of the art platforms can be applied to unravel the dynamics of the BCR repertoire. Several tools and softwares, equipped with V(D)J gene alignment, CDR3 sequence identification, mutation frequencies, have been developed for BCR sequence analysis, and are described in detail elsewhere (53, 54). The analysis of BCR heavy-chain genes can be performed on bulk B cells (55), specific B cell subsets (56) or single-cell (25). Data allow to track and characterize the molecular phenotype and clonal evolution of spike-specific MBC clones from early time points after vaccine administration to longer time points. The approach can be instrumental for highlighting differences between responses to natural infection and vaccination (55) and for comparing different vaccine platforms (57). Recently, the BCR repertoire approach has been employed to study the RBD-specific MBCs affinity toward multiple viral variants (25, 30).

## Conclusions

After about 20 months from the beginning of the vaccination campaign, milestones in the knowledge of the immunological mechanisms underlying immune responsiveness to SARS-CoV-2 vaccination have been reached, although many different factors can impact on the analysis. Moreover, the overlapping of vaccination and infection, contracted before, during or after the last vaccine dose, has altered the dynamics and the magnitude of the response, and open questions remain on how the immune system will respond on its next encounter with the virus, and how the first exposure, whether it was vaccination or infection, may influence the response. Some studies have robustly correlated the SARS-CoV-2 vaccination efficacy with *in vitro* neutralizing and binding antibodies, supporting the use of post-immunization antibody titers as the basis for establishing a correlate of protection for COVID-19 vaccines (14, 58). Nevertheless, considering the physiological decline of circulating antibodies overtime, the lack of a threshold value for protection from infection, and the rapid appearance of novel viral variants, the measurement of MBC can be evaluated as an indicator of potentially antibody-producing cells upon antigen encounter.

Therefore, beyond the follow-up of the antibody response, it has become evident that knowledge of the B cell response, that is the cellular biology underpinning the antibody response, is crucial for understanding how vaccinated people will respond to repeated booster doses or infection. Different technologies aimed to identify, quantify, and measure the spike or RBD specific B cells, monitor their persistence, their evolution, their functionality upon antigen encounter, are available for researchers to deeply profile the B cell response adding fundamental notions to the comprehension of the vaccine elicited immune response.

## Author contributions

EP, DM, and AC conceived and wrote the manuscript. All authors contributed to the article and approved the submitted version.

## Funding

This study was supported by the Department of Medical Biotechnologies of the University of Siena. Publication costs were supported by University of Siena.

## Conflict of interest

The authors declare that the research was conducted in the absence of any commercial or financial relationships that could be construed as a potential conflict of interest.

## Publisher’s note

All claims expressed in this article are solely those of the authors and do not necessarily represent those of their affiliated organizations, or those of the publisher, the editors and the reviewers. Any product that may be evaluated in this article, or claim that may be made by its manufacturer, is not guaranteed or endorsed by the publisher.
